# Comparison of lumbar endoscopic unilateral laminotomy bilateral decompression and minimally invasive surgery transforaminal lumbar interbody fusion for one-level lumbar spinal stenosis

**DOI:** 10.1186/s12891-020-03820-2

**Published:** 2020-11-27

**Authors:** Wenbin Hua, Bingjin Wang, Wencan Ke, Xinghuo Wu, Yukun Zhang, Shuai Li, Shuhua Yang, Cao Yang

**Affiliations:** Department of Orthopaedics, Union Hospital, Tongji Medical College, Huazhong University of Science and Technology, Wuhan, 430022 China

**Keywords:** Endoscopic, Unilateral laminotomy bilateral decompression, Minimally invasive surgery, Transforaminal lumbar interbody fusion, Lumbar spinal stenosis

## Abstract

**Background:**

The aim of the present study is to compare the clinical outcomes and postoperative complications of lumbar endoscopic unilateral laminotomy bilateral decompression (LE-ULBD) and minimally invasive surgery transforaminal lumbar interbody fusion (MIS-TLIF) to treat one-level lumbar spinal stenosis (LSS) without degenerative spondylolisthesis or deformity.

**Methods:**

A retrospective analysis of 112 consecutive patients of one-level LSS undergoing either LE-ULBD or MIS-TLIF was performed. Patient demographics, operation time, estimated blood loss, time to ambulation, length of hospitalization, intraoperative and postoperative complications were recorded. The visual analog scale (VAS) score for leg and back pain, the Oswestry Disability Index (ODI) score, and the Macnab criteria were used to evaluate the clinical outcomes. The healthcare cost was also recorded.

**Results:**

The operation time, estimated blood loss, time to ambulation and length of hospitalization of LE-ULBD group were shorter than MIS-TLIF group. The postoperative mean VAS and ODI scores decreased significantly in both groups. According to the modified Macnab criteria, the outcomes rated as excellent/good rate were 90.6 and 93.8% in the two groups. The mean VAS scores, ODI scores and outcomes of the modified Macnab criteria of both groups were of no significant difference. The healthcare cost of LE-ULBD group was lower than MIS-TLIF group. Two cases of intraoperative epineurium injury were observed in the LE-ULBD group. One case of cauda equina injury was observed in the LE-ULBD group. No nerve injury, dural injury or cauda equina syndrome was observed in MIS-TLIF group. However, one case with transient urinary retention, one case with pleural effusion, one case with incision infection and one case with implant dislodgement were observed in MIS-TLIF group.

**Conclusions:**

Both LE-ULBD and MIS-TLIF are safe and effective to treat one-level LSS without degenerative spondylolisthesis or deformity. LE-ULBD is a more minimally invasive option and of less economic burden compared with MIS-TLIF. Decompression plus instrumented fusion may be not necessary for one-level LSS without degenerative spondylolisthesis or deformity.

## Background

Lumbar spinal stenosis (LSS) can be caused by degenerative facet joints, hypertrophic ligamentum flavum, bulging or protrusion of the intervertebral discs, spondylolisthesis and or a combination of the above pathological lesions [1, 2]. These pathological lesions may cause radicular leg pain and neurogenic claudication [1, 3, 4]. Surgery may be necessary to relieve the symptoms and improve function after failed conservative treatment in patients with LSS [1, 3, 5].

Microsurgical procedures was firstly described by Yasargil and Caspar in 1977 [6]. Microscopic laminotomy and foraminotomy remains the gold standard for the decompression of LSS [7, 8]. Minimally invasive unilateral laminotomy bilateral decompression was performed to treat LSS with less injury to the paraspinal musculoligamentous structures [4, 9]. To further minimize injury to the paraspinal musculoligamentous structures, lumbar endoscopic unilateral laminotomy bilateral decompression (LE-ULBD) has been used to treat LSS in recent years [4, 8, 10, 11].

Laminotomy with medial facetectomy may result in segmental spinal instability, instrumented fusion may be necessary in some cases to prevent segmental spinal instability after decompression of LSS [12, 13]. Transforaminal lumbar interbody fusion (TLIF) has been performed to treat LSS since 1980s [14]. Minimally invasive surgery transforaminal lumbar interbody fusion (MIS-TLIF) was firstly described by Foley et al. and had been commonly performed to treat LSS with minimized injury to the paraspinal musculoligamentous structures [15–17].

Even though both decompression alone and decompression plus instrumented fusion has been performed to treat patients with LSS, it is controversial about the necessity of instrumented fusion after decompression [8, 12, 18, 19]. Besides, degenerative spondylolisthesis and scoliosis are two associated conditions which may determine the surgical management of LSS. The purpose of the present retrospective study is to compare the clinical effect, safety and complications of LE-ULBD and MIS-TLIF to treat one-level LSS without degenerative spondylolisthesis or deformity.

## Methods

### Patient population and grouping

This retrospective study included 112 patients (44 males, 68 females) with one-level LSS, who underwent LE-ULBD or MIS-TLIF in our department between January 2016 and December 2017. The present study was conducted in accordance with the guidelines of the Declaration of Helsinki and was approved by the ethics committee of our College. Written informed consents was obtained from each patient. In these patients, 32 surgeries were performed by LE-ULBD and the other 80 surgeries were performed by MIS-TLIF.

Inclusion criteria were as follows: patients with (1) main symptoms, including leg pain, numbness, motor weakness or neurogenic claudication; (2) computed tomography and/or magnetic resonance imaging indicating central stenosis and/or lateral recess stenosis, in agreement with clinical symptoms and signs; (3) a history of failed conservative treatment, including physical therapy and nonsteroidal anti-inflammatory drugs intervention for more than 3 months; and (4) follow-up for at least 24 months. Exclusion criteria were as follows: patients with (1) degenerative spondylolisthesis or deformity; (2) tumors, infections, or other lesions; and (3) a surgical history involving the corresponding segment [4, 20]. Each patient that met all the criteria underwent either LE-ULBD or MIS-TLIF under general anesthesia. The surgery procedures were chosen according to preoperative conversation, a variety of patient factors and surgeon preference.

### Surgical technique

All the surgeries were performed by the senior author, who has many years of experience in open and minimally invasive lumbar canal decompression, and lumbar endoscopic visualized discectomy. Both LE-ULBD and MIS-TLIF were performed under general anesthesia with the patient in the prone position.

### LE-ULBD

Posteroanterior and lateral fluoroscopy were used to locate the interlaminar space at the surgery segment. A 10 mm skin incision was made laterally to the outer border of the interlaminar window. Soft tissue expanders were applied via the incision in order to separate the muscles to allow the insertion of the working sheath and the endoscopic surgical system (working channel 4.3 mm, outside diameter 7.0 mm, working length 130.0 mm, lens angle 30 degrees). All the subsequent procedures were performed under constant irrigation with excellent endoscopic visualization.

**Fig. 1:**
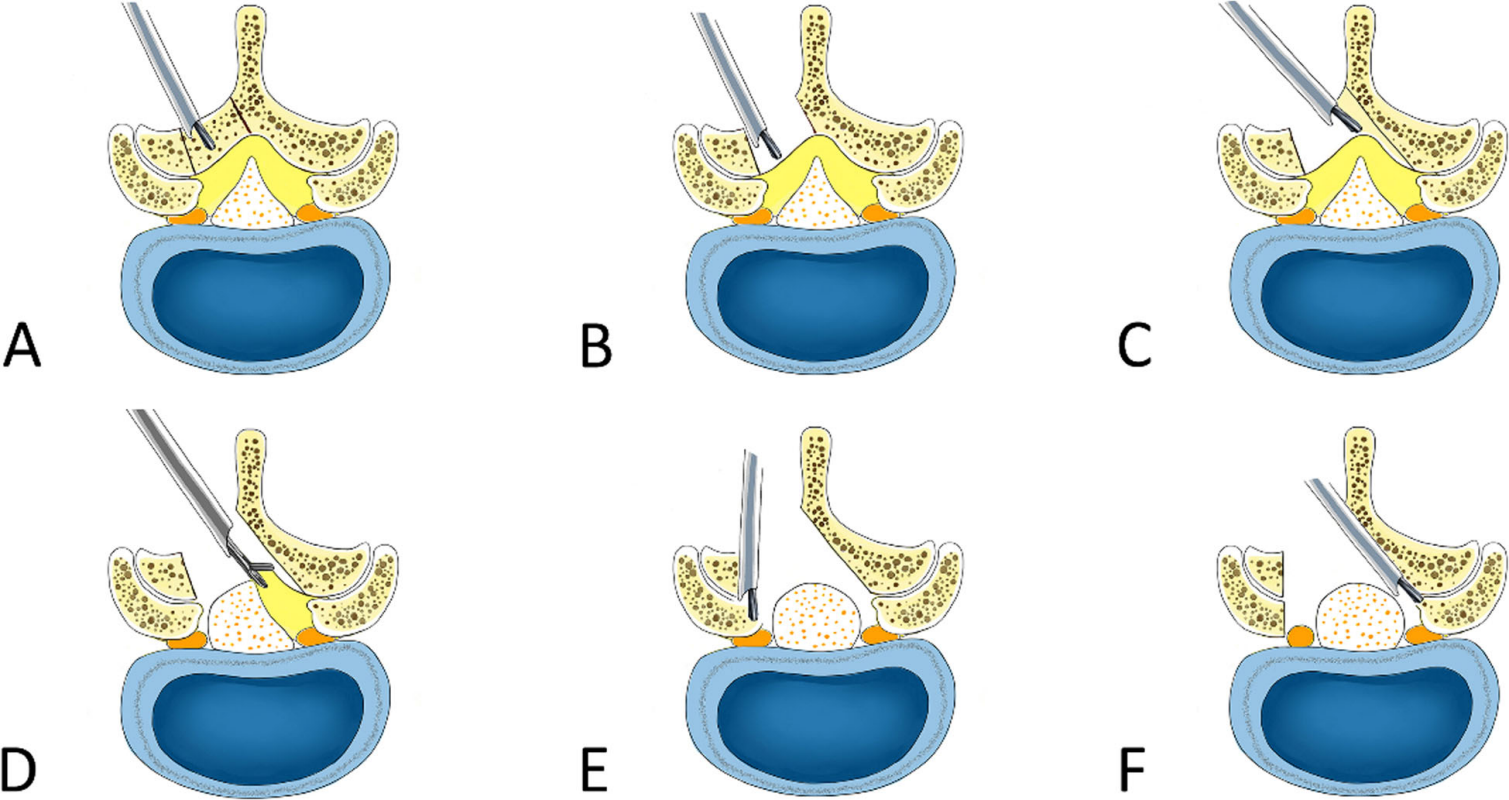
Surgical procedures of lumbar endoscopic unilateral laminotomy bilateral decompression. **a** and **b** The inferior edge of the cranial lamina and the base of the spinous process of the ipsilateral side were removed by the endoscopic burr; **c** undercutting of the contralateral cranial lamina was performed; **d** the ipsilateral and contralateral ligamentum flavum was identified and removed piecemeal with endoscopic punches and forceps; **e** the ipsilateral medial facetectomy was performed to decompress the lateral recess and foramen, ensuring adequate decompression of the traversing nerve root (**f**) the contralateral medial facetectomy was performed to decompress the lateral recess and foramen, ensuring adequate decompression of the traversing nerve root

**Fig. 2:**
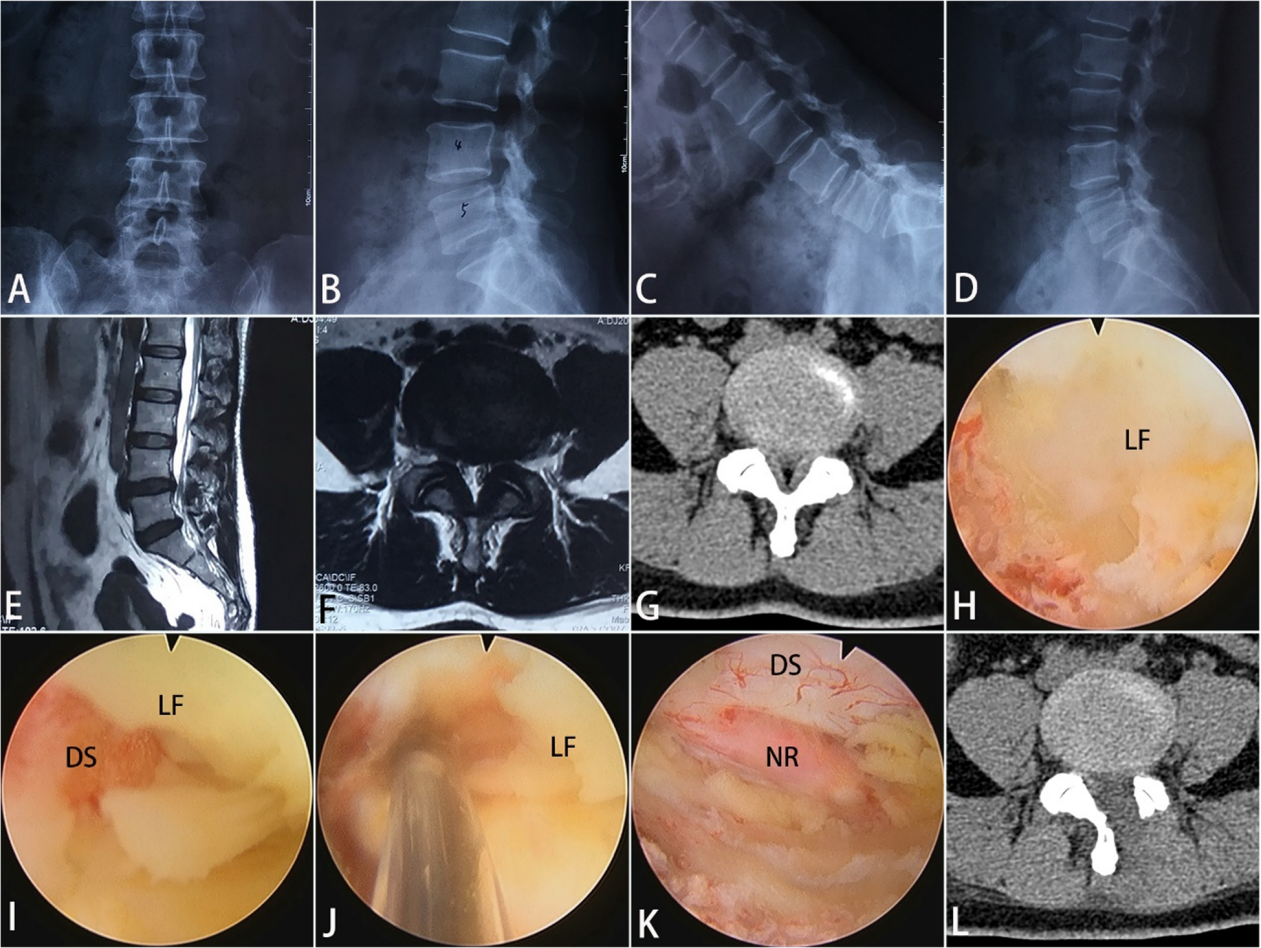
Lumbar endoscopic unilateral laminotomy bilateral decompression performed on a 51-year-old female patient diagnosed with L4-L5 lumbar spinal stenosis without degenerative spondylolisthesis or deformity. **a** and **b** preoperative anteroposterior and lateral plain radiographs; **c** and **d** preoperative flexion and extension radiographs; **e** and **f** preoperative magnetic resonance imaging scans; **g** preoperative computed tomography (CT) scans; **h** undercutting of the ipsilateral and contralateral cranial lamina was performed; **i** the ipsilateral and contralateral ligamentum flavum (LF) was identified and removed piecemeal with endoscopic punches and forceps; **j** the bipolar radiofrequency electrocoagulator was used for hemostasis; **k** adequate decompression of the ipsilateral and contralateral traversing nerve root (NR) and the dural sac (DS); **l** CT scans one week after the surgery

The inferior edge of the cranial lamina and the base of the spinous process of the ipsilateral side were removed by the endoscopic burr, enabling access into the spinal canal. Once the epidural space was entered, undercutting of the contralateral cranial lamina was performed. Then the ipsilateral and contralateral ligamentum flavum was identified and removed piecemeal with endoscopic punches and forceps. Subsequently, ipsilateral and contralateral medial facetectomy was performed to decompress the lateral recess and foramen, ensuring adequate decompression of the traversing nerve root. Once the traversing nerve root was decompressed, it was reflected medially using a blunt dissector. These procedures were presented in Fig. 1. The bipolar radiofrequency electrocoagulator was used for hemostasis, soft-tissue clearance and adhesion release. Prior to surgery completion, we ensured there was no significant dural sac damage or active bleeding. No drainages were required. A representative case is shown in Fig. 2.

### MIS-TLIF

**Fig. 3:**
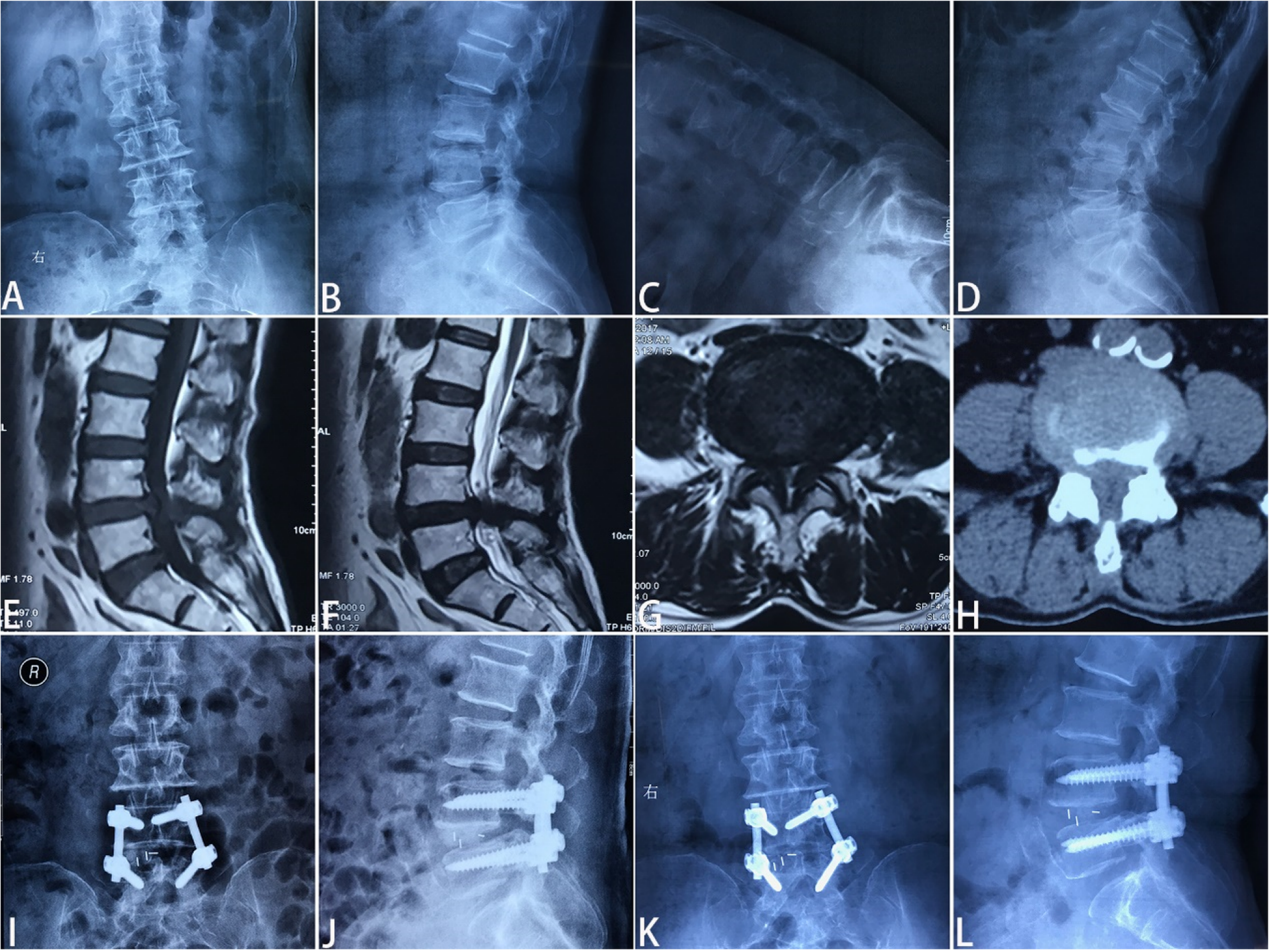
Minimally invasive surgery transforaminal lumbar interbody fusion performed on a 70-year-old female patient diagnosed with L4-L5 lumbar spinal stenosis without degenerative spondylolisthesis or deformity. **a** and **b** preoperative anteroposterior and lateral plain radiographs; **c** and **d** preoperative flexion and extension radiographs; **e-g** preoperative magnetic resonance imaging scans; **h** preoperative computed tomography (CT) scans; **i** and **j** postoperative anteroposterior and lateral plain radiographs; **i** anteroposterior and lateral plain radiograph 6 months after the surgery

Posteroanterior and lateral fluoroscopy were used to locate the pedicles of the surgery level. The Wiltse approach was undertaken through a paramedian skin incision. A Quadrant tubular dilator was used for unilateral facet exposure. Facetectomy was performed on the ipsilateral side in order to visualize the transforaminal disc space. Laminectomy and lateral recess decompression were performed to decompress the spinal canal. Besides, the tubular retractor could be angled medially to complete a more extensive decompression of central canal stenosis and the contralateral side [16]. Ligamentum flavum was adequately resected to expose the ipsilateral traversing and exiting nerve roots. A standard discectomy and endplates removal were performed to allow for an intervertebral cage insertion [16]. The autogenous and allogeneic bone graft was placed anteriorly and contralateral to the annulotomy, then an intervertebral cage filled with autogenous and allogeneic bone graft was placed. In addition, unilateral pedicle screws were placed ipsilateral to the approach, and contralateral pedicle screws were placed through a contralateral incision. Rods were sized appropriately and subfascially tunneled through the paramedian incisions. The incisions were irrigated and closed in layers with drainages kept for no more than 48 h. A representative case is shown in Fig. 3.

### Clinical evaluation

The operation time, estimated blood loss, time to ambulation, length of hospitalization, Intraoperative and postoperative complications were recorded. Follow-up examinations were conducted at 3, 6, 12 and 24 months postoperatively. Postoperative magnetic resonance imaging or computed tomography imaging was performed when necessary. The visual analog scale (VAS) score for leg pain and back pain (range, 0–10), Oswestry Disability Index (ODI) score (range, 0–100) and modified Macnab criteria were recorded preoperatively and at follow-up postoperatively. The surgery cost, anesthesia cost, surgical equipment and medical materials cost was also recorded.

### Statistical analyses

All data are presented as mean ± standard deviation. SPSS 22.0 (IBM Corp., Armonk, NY, USA) was used to perform the statistical analyses. GraphPad Prism 6 (Graph Pad Software, Inc., San Diego, CA, USA) was used to generate plots. Nonparametric statistical analysis, including Mann-Whitney U test or Wilcoxon signed-rank test were used. A *p*-value of less than 0.05 was considered statistically significant.

## Results

### Demographic data

The clinical characteristics of both groups were summarized in Table 1. The mean duration of the main symptoms was 5.1 ± 1.8 months, ranging from 3 to 60 months.

**Table 1:** Baseline Characteristics of the Two Groups

	LE-ULBD	MIS-TLIF	*P* value
N	32	80	–
Male/ Female	12/20	32/48	0.808
Age (years)	56.7 ± 9.1 (28–77)	58.8 ± 10.5 (39–85)	0.515
Levels involved			
L2-L3	0 (0%)	2 (2.5%)	0.652
L3-L4	3 (9.4%)	2 (2.5%)	
L4-L5	20 (62.5%)	52 (65%)	
L5-S1	9 (28.1%)	24 (30%)	
Preoperative symptoms			
Back pain	29 (90.6%)	71 (88.8%)	0.777
Leg pain	28 (87.5%)	70 (87.5%)	1.000
Numbness	26 (81.2%)	67 (83.8%)	0.751
Motor weakness	21 (65.6%)	58 (72.5%)	0.473

*N indicates number of patients included in the statistical analysis, LE-ULBD* lumbar endoscopic unilateral laminotomy bilateral decompression, *MIS-TLIF* minimally invasive surgery transforaminal lumbar interbody fusion

### Clinical outcomes

**Fig. 4:**
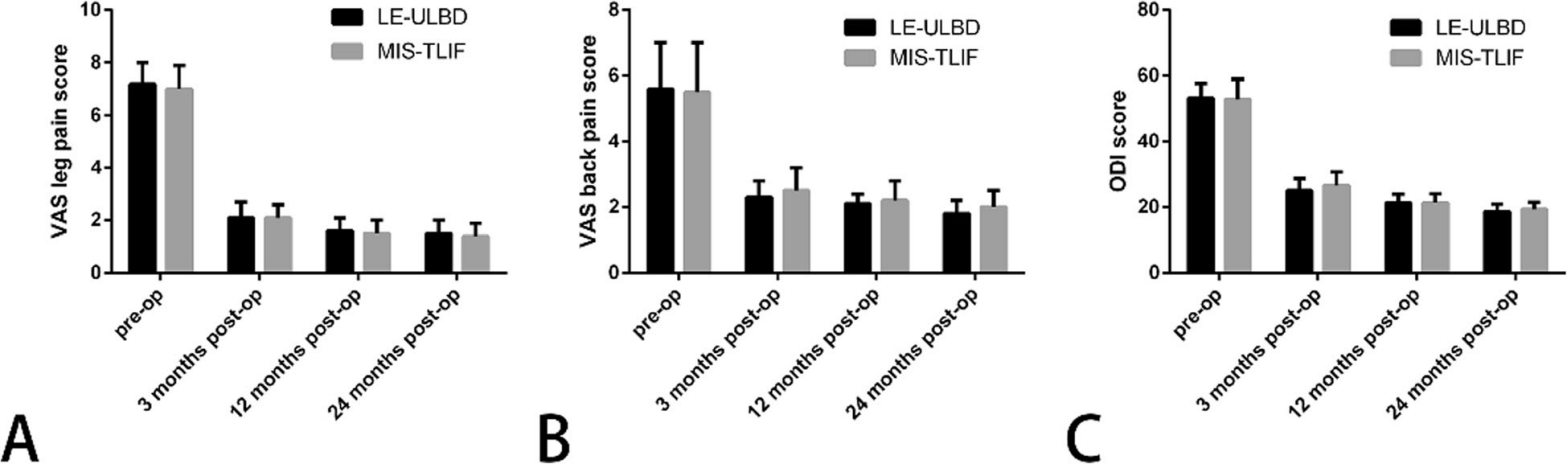
The mean visual analog scale (VAS) scores for leg and back pain, and Oswestry disability index (ODI) scores. **a** VAS scores for leg pain; **b** VAS scores for back pain; **c** ODI scores. Pre-op, preoperative; post-op, postoperative; LE-ULBD, lumbar endoscopic unilateral laminotomy bilateral decompression; MIS-TLIF, minimally invasive surgery transforaminal lumbar interbody fusion

The operation time, estimated blood loss, time to ambulation and length of hospitalization of LE-ULBD group were shorter than MIS-TLIF group (Table 2). The mean VAS scores and ODI scores improved significantly postoperatively in both LE-ULBD and MIS-TLIF groups (Table 3). According to the modified Macnab criteria, the outcomes rated as excellent/good rate were 90.6 and 93.8% in the two groups (Table 4). The mean VAS scores, ODI scores and outcomes of the modified Macnab criteria of both groups were of no significant difference (Fig. 4).

**Table 2:** Clinical Outcomes and Complications of the Two Groups

	LE-ULBD	MIS-TLIF	*P* value
N	32	80	–
Operation time (min)	139.5 ± 31.2 (85–240)	161.1 ± 45.6 (60–300)	0.023
Estimated blood loss (ml)	51.9 ± 10.9 (40–80)	146.6 ± 80.3 (50–400)	< 0.001
Time to ambulation (h)	11.7 ± 3.6 (8–28)	22.1 ± 9.5 (12–48)	< 0.001
Length of hospitalization (d)	2.7 ± 0.9 (1–4)	11.2 ± 2.4 (7–17)	< 0.001

*N indicates number of patients included in the statistical analysis, LE-ULBD* lumbar endoscopic unilateral laminotomy bilateral decompression, *MIS-TLIF* minimally invasive surgery transforaminal lumbar interbody fusion

**Table 3:** Comparison of VAS and ODI Scores in the Two Groups

		LE-ULBD	MIS-TLIF	*P* value
N		32	80	–
VAS leg pain	Pre-op	7.2 ± 0.8	7.0 ± 0.9	0.655
	3 months Post-op	2.1 ± 0.6*	2.1 ± 0.5*	0.847
	12 months Post-op	1.6 ± 0.5*	1.5 ± 0.5*	0.061
	24 months Post-op	1.5 ± 0.5*	1.4 ± 0.5*	0.252
VAS back pain	Pre-op	5.6 ± 1.4	5.5 ± 1.5	0.672
	3 months Post-op	2.3 ± 0.5*	2.5 ± 0.7*	0.249
	12 months Post-op	2.1 ± 0.3*	2.2 ± 0.6*	0.335
	24 months Post-op	1.8 ± 0.4*	2.0 ± 0.5*	0.328
ODI (%)	Pre-op	53.2 ± 4.6	52.9 ± 6.2	0.767
	3 months Post-op	25.1 ± 3.7*	26.6 ± 4.2*	0.126
	12 months Post-op	21.4 ± 2.6*	21.3 ± 2.8*	0.780
	24 months Post-op	18.8 ± 2.1*	19.4 ± 2.1*	0.212

*N indicates number of patients included in the statistical analysis, LE-ULBD* lumbar endoscopic unilateral laminotomy bilateral decompression, *MIS-TLIF* minimally invasive surgery transforaminal lumbar interbody fusion, *Pre-op* preoperative*, Post-op* postoperative*, VAS* Visual Analog Scale, *ODI* Oswestry Disability Index. *P < 0.05 versus *preoperative data*

**Table 4:** Comparison of Modified Macnab Evaluation in the Two Groups

Modified Macnab evaluation		LE-ULBD	MIS-TLIF	*P* value
Outcome	Excellence (N)	22	51	NS
	Good (N)	7	24	NS
	Fair (N)	3	5	NS
	Poor (N)	0	0	NS
Excellence/good rate (%)		90.6	93.8	NS

*N indicates number of patients included in the statistical analysis, LE-ULBD* lumbar endoscopic unilateral laminotomy bilateral decompression, *MIS-TLIF* minimally invasive surgery transforaminal lumbar interbody fusion, *NS* not significant

### Complications

Intraoperative and postoperative complications of LE-ULBD and MIS-TLIF group were also compared (Table 5). Two cases of intraoperative epineurium injury were observed in the LE-ULBD group. One case of cauda equina injury was observed in the LE-ULBD group. This case was presented with foot dorsiflexion and defecation dysfunction and recovered within 6 months. No nerve injury, dural injury or cauda equina syndrome was observed in MIS-TLIF group. However, one case with transient urinary retention, one case with pleural effusion, one case with incision fat liquefaction and one case with implant dislodgement were observed in MIS-TLIF group. No reoperation was observed within 90 days or during 24 months of follow-up.

**Table 5:** Complications of the Two Groups

	LE-ULBD	MIS-TLIF	*P* value
N	32	80	–
Intraoperative complications			
Dural tears	2 (6.2%)	0 (0%)	0.025
Cauda equina injury	1 (3.1%)	0 (0%)	0.114
Intraoperative complication rate	3 (9.4%)	0 (0%)	0.006
Postoperative complications			
Transient urinary retention	0 (0%)	1 (1.2%)	0.527
Pleural effusion	0 (0%)	1 (1.2%)	0.527
Incision fat liquefaction	0 (0%)	1 (1.2%)	0.527
Incision infection	0 (0%)	0 (0%)	1.000
Implant dislodgement	–	1 (1.2%)	–
Postoperative complication rate	0 (0%)	4 (5.0%)	0.200

*N indicates number of patients included in the statistical analysis, LE-ULBD* lumbar endoscopic unilateral laminotomy bilateral decompression, *MIS-TLIF* minimally invasive surgery transforaminal lumbar interbody fusion

### Healthcare cost

The surgery cost, anesthesia cost, surgical equipment and medical materials cost of LE-ULBD group were less than MIS-TLIF group (Table 6).

**Table 6:** Healthcare Cost of the Two Groups

	LE-ULBD	MIS-TLIF	*P* value
N	32	80	–
Surgery cost	7125.4 ± 852.3 (6221–8256)	10,479.5 ± 1100.6 (8999–14,359)	< 0.001
Anesthesia cost	3609.2 ± 388.6 (3359–5424)	3855.5 ± 311.4 (3207–4259)	< 0.001
Surgical equipment and medical materials cost	3900.0 ± 0.0 (3900–3900)	41,864.2 ± 7684.8 (33333–54,245)	< 0.001

*N indicates number of patients included in the statistical analysis, LE-ULBD* lumbar endoscopic unilateral laminotomy bilateral decompression, *MIS-TLIF* minimally invasive surgery transforaminal lumbar interbody fusion; healthcare cost was compared in China Yuan (CNY)

## Discussion

LSS is traditionally treated with open or microscopic laminotomy and foraminotomy via a midline lumbar incision [21]. However, extensive detaching the paraspinal muscles from the spinous processes and lamina may cause increased intraoperative blood loss, postoperative pain and weakness secondary to muscle denervation [21]. Besides, supraspinous and interspinous ligaments injury and extensive facetectomy may cause iatrogenic spinal instability, requiring additional posterior fixation for stabilization [4, 21, 22]. Therefore, various minimally invasive techniques were developed to minimize the surgical trauma [7, 9, 23]. Patients with neurogenic claudication secondary to LSS without degenerative spondylolisthesis or deformity could be treated by both decompression alone and decompression with instrumented fusion [12, 18, 19]. LE-ULBD and MIS-TLIF are two common minimally invasive procedures to treat LSS, and typical representative of decompression alone technique and decompression plus instrumented fusion technique, respectively. The development of endoscopic spine surgery has been evolving rapidly, therefore it is feasible to perform interlaminar decompression via both LE-ULBD and MIS-TLIF [20]. The present retrospective study revealed that both LE-ULBD and MIS-TLIF are effective to treat one-level LSS without degenerative spondylolisthesis or deformity. While, LE-ULBD is a more minimally invasive option for patients with one-level LSS without degenerative spondylolisthesis or deformity. Moreover, LE-ULBD is of less economic burden than MIS-TLIF.

During LE-ULBD, laminotomy and and foraminotomy could be safely performed under excellent endoscopic visualization to guarantee complete decompression, minimize surgical trauma, and prevent spinal instability [8, 10, 11]. However, sufficient decompression without violating the stability of the facet joints may be technically difficult in cases with narrow interlaminar spaces, posterior marginal osteoproliferation of the vertebrae, ossification of the posterior longitudinal ligaments, and recurrences [8, 10]. Excessive facetectomy may be inevitable for sufficient lateral recess decompression and foraminotomy, exacerbating postoperative instability. In the present study, undercutting of the cranial lamina was performed during LE-ULBD to overcome the difficulty during insertion of the working sheath [4]. Additionally, the excellent endoscopic visualization achieved during LE-ULBD ensured the undercutting of the cranial lamina, minimized facetectomy, and sufficient decompression of the lateral recess and foramen.

The advantages of LE-ULBD were to perform bilateral decompression via a unilateral approach with minimize traumatization to the paraspinal musculoligamentous structures; to ensure the sufficient decompression the lateral recess and foramen under excellent endoscopic visualization to minimize neurological injury; and to preserve the stability of the spine with minimized foraminotomy [4, 8, 10, 11]. On the other hand, LE-ULBD has some disadvantages, such as the steep learning curve. Muscles, facet cysts, and ligaments may be difficult to identify under endoscopic visualization, increasing the risk for iatrogenic injury.

MIS-TLIF has been demonstrated to be a safe option for lumbar fusion with minimized iatrogenic traumatization to the paraspinal musculoligamentous structures [16, 17]. Compared with traditional open TLIF, MIS-TLIF was of similar good clinical outcomes, fusion rates, less postoperative back pain, shorter time to ambulation, and length of hospitalization [16, 17]. MIS-TLIF was performed to achieve the sufficient decompression of LSS, immediate improvement of spinal alignment, and prevention of spinal instability.

While it is controversial about the necessity of instrumented fusion after decompression of LSS [8, 12, 18, 19]. Nowadays, there are growing evidences suggest that decompression alone is better for LSS without degenerative spondylolisthesis or deformity [24]. Försth et al. [19, 25] found that decompression with instrumented fusion did not result in better clinical outcomes than decompression alone for LSS with or without degenerative spondylolisthesis. Compared with decompression alone surgery, the addition of instrumented fusion to decompression surgery significantly increased the hospital costs, including the costs of surgery and the length of hospitalization [19]. In the present study, both LE-ULBD and MIS-TLIF were of excellent outcomes for LSS without degenerative spondylolisthesis or deformity, as a result, addition of instrumented fusion to decompression surgery may be not necessary. Therefore, LSS without degenerative spondylolisthesis or deformity could be treated by LE-ULBD. Moreover, endoscopic decompression is of higher technical demand and steep learning curve.

There are some limitations to the present study. First, it is a retrospective, non-randomized controlled cohort study with a small sample size and short follow-up period. There also may be selection bias, as surgeons determined whether decompression alone or decompress with instrumented fusion should be performed. Further prospective, randomized, controlled studies, with larger sample sizes and longer follow-up periods should be conducted to determine the optimal surgical management for patients with LSS. Second, all the patients included were of one-level LSS without degenerative spondylolisthesis or deformity. As a result, further studies should be conducted to compare the clinical outcomes of LE-ULBD and MIS-TLIF for LSS with degenerative spondylolisthesis or deformity. Besides, preexisting adjacent level degeneration was not evaluated and compared in the present study.

## Conclusion

Both LE-ULBD and MIS-TLIF are safe and effective to treat one-level LSS without degenerative spondylolisthesis or deformity. LE-ULBD was proved to be a more minimally option and of less economic burden compared with MIS-TLIF. Decompression with instrumented fusion may be not necessary for one-level LSS without degenerative spondylolisthesis or deformity.

## Data Availability

The data sets supporting the conclusion of this article are included in the manuscript. Upon request, raw data can be provided by the corresponding author.

## Abbreviations

LE-ULBD: Lumbar endoscopic unilateral laminotomy bilateral decompression
MIS-TLIF: Minimally invasive surgery transforaminal lumbar interbody fusion
LSS: Lumbar spinal stenosis
VAS: Visual analog scale
ODI: Oswestry Disability Index.
